# A Rare Cause of Uveitis: Vemurafenib

**DOI:** 10.4274/tjo.95914

**Published:** 2018-12-27

**Authors:** Selçuk Sızmaz, Nuhkan Görkemli, Ebru Esen, Nihal Demircan

**Affiliations:** 1 Çukurova University Faculty of Medicine, Department of Ophthalmology, Adana, Turkey

**Keywords:** Drug-induced uveitis, melanoma, vemurafenib

## Abstract

A 25-year-old female presented with a decrease of vision and redness in both eyes. She had a history of nodular melanoma in her right shoulder, which was excised surgically and she was under oral vemurafenib treatment. She was diagnosed with moderately severe bilateral panuveitis and hospitalized for systemic investigation and workup. The laboratory test results were unremarkable and systemic workup failed to reveal an etiology. The condition was considered vemurafenib-induced uveitis, as the drug is known to be associated with uveitis. After reevaluation with the oncology department, vemurafenib was stopped and topical and systemic corticosteroid therapy was started. The uveitis resolved and her vision returned to normal. No sign of recurrence was detected at 8-month follow-up.

## Introduction

Various drugs, regardless of the route of administration, have been reported to be associated with uveitis, an entity referred to as drug-induced uveitis. While many more drugs have been implicated, the most commonly reported are cidofovir, rifabutin, bisphosphonates, sulfonamides, tumor necrosis factor inhibitors, and fluoroquinolones. The underlying mechanism is proposed to be either inflammatory or toxic.^1,2^ Hence, when managing a uveitis patient, the physician should always keep in mind that a drug could be involved in the etiology.

Vemurafenib (Zelboraf, F. Hoffmann-La Roche Ltd, Basel, Switzerland), a potent oral BRAF^V600^ inhibitor, is a new drug shown to be effective against advanced cutaneous melanoma.^3^ The drug was found to be more effective than dacarbazine in reducing mortality risk.^4^ Reported systemic adverse effects of vemurafenib are arthralgia (53%), alopecia (45%), fatigue (38%), nausea (35%), and photosensitivity (33%).^5^ In addition, uveitis was the most commonly reported adverse event related to vemurafenib administration, followed by conjunctivitis and dry eye (4%, 2.8%, and 2%, respectively).^3^ Adverse events must be weighed against the potential survival benefit. When it comes to uveitis, steroids were reported to suppress ocular inflammation; however, some authors were in favor of cessation of the therapy.^5^

The aim of this study was to present a case of vemurafenib-induced panuveitis.

## Case Report

A 25-year-old woman presented with diminished vision and redness in both eyes. She had a history of resected nodular melanoma in her right shoulder and was under vemurafenib therapy (960 mg/day) initiated at another center, though her family history was unremarkable.

**Figure 1:**
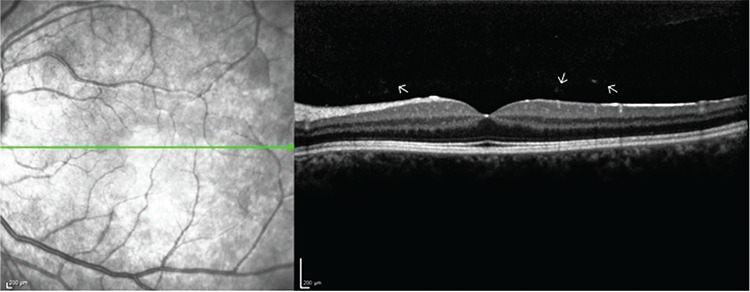
Optical coherence tomography of the posterior pole. Note the clumps of cells in the posterior vitreous showing hyperreflectivity (arrows)

She had 20/200 visual acuity in her right eye which did not improve with correction. Corrected visual acuity was 20/20 in her left eye. Both eyes had normal intraocular pressure readings. Slit-lamp biomicroscopy revealed bilateral 2-3+ cells in the anterior chamber, posterior synechia, and pigment precipitates on the lens, all of which were more severe in the right eye. The fundus was not clear in the right eye due to cells in the vitreous. There were vitreous cells in the left eye; however, the optic nerve, macula, and the peripheral retina seemed normal. On fluorescein angiography, the right eye could not be visualized due to vitreal inflammation; the left eye was normal except peripheral vascular leakage in the late phases of the angiogram. The right eye could not be visualized on optical coherence tomography either; however, in the left eye the retina was normal, with clumps of cells in the posterior vitreous (Figure 1). The patient was hospitalized for investigation with the diagnosis of bilateral panuveitis.

The results of diagnostic tests investigating possible etiologies were unremarkable. Systemic workup also failed to lead to a specific diagnosis.

When the patient was questioned in more detail regarding her history, she reported she had had similar symptoms in the past which resolved with cessation of vemurafenib therapy. The patient was evaluated in the oncology department of our hospital and they suggested discontinuing vemurafenib. Oral prednisone 1 mg/kg, topical prednisolone acetate (hourly) and cycloplegic drops (three times daily) were started for both eyes.

Her inflammatory findings subsided and the systemic and topical steroids were tapered. She has been under follow-up for 8 months and has exhibited no signs of recurrence of the uveitis or melanoma.

## Discussion

Herein, we report a case of bilateral vemurafenib-induced uveitis. No uveitis was reported in a phase 3 trial of vemurafenib.^6^ However, uveitis as an adverse effect of vemurafenib was published in several case reports.^7^ Therefore, albeit rare, it is a noteworthy side effect of treatment.

Several mechanisms are believed to be involved in the pathogenesis of vemurafenib-induced uveitis. One is the result of vemurafenib-induced lymphocytic infiltration of subclinical uveal metastases. The second is a possible inflammatory response to antigens shared by melanocytes in the melanoma and the choroid.^3,5^ The period between the first administration of vemurafenib treatment and initial uveitis symptoms was reported to vary from 1 to 85 weeks (average of 27).^5^

We attributed uveitis to vemurafenib, as we could detect no potential cause in spite of a thorough etiologic investigation and systemic workup. Moreover, the findings resolved with the cessation of the drug and no recurrence was encountered during a period of 8 months.

Our patient had bilateral panuveitis. Guedj et al.^7^ reported 7 cases of bilateral vemurafenib-induced uveitis in patients aged 69-81. Six of their patients had anterior uveitis and one had severe panuveitis. Our patient was very young compared to theirs, and the youngest patient in the literature reported to have vemurafenib-induced uveitis. The course of uveitis might be less severe in the elderly.

Whether to discontinue treatment in cases of drug-induced uveitis is controversial. This should be decided based on each patient’s ocular findings and systemic condition, in collaboration with oncologists. In a recent report, Fierz et al.^8^ continued vemurafenib despite bilateral anterior uveitis which could be controlled with topical steroids. In one of the very first reports of vemurafenib-induced uveitis, the drug was stopped and the uveitis was controlled with systemic steroid therapy.^9^ This was similar to our case.

In conclusion, uveitis is not an uncommon adverse effect of vemurafenib, a successful therapeutic option in metastatic melanoma. As vemurafenib becomes more widely used, clinical variation in cases of uveitis associated with its use will also increase. Mild cases can be controlled with topical steroids; however, severe cases could require cessation of treatment-despite the risk of worsening of the systemic disease-and systemic steroid therapy.
